# Biomechanical comparison of three stand-alone lumbar cages — a three-dimensional finite element analysis

**DOI:** 10.1186/1471-2474-14-281

**Published:** 2013-10-02

**Authors:** Shih-Hao Chen, Ming-Chieh Chiang, Jin-Fu Lin, Shang-Chih Lin, Ching-Hua Hung

**Affiliations:** 1 Department of Orthopedics, Tzu-Chi General Hospital at Taichung and Tzu Chi University, Taichung, Taiwan; 2 Department of Mechanical Engineering, National Chiao Tung University, 1001 University Road, Hsinchu 30010, Taiwan; 3 BoneCare Orthopedic Centers, Han-Chiung Clinics, Taipei, Taiwan; 4 Graduate Institute of Biomedical Engineering, National Taiwan University of Science and Technology, Taipei, Taiwan

**Keywords:** ALIF, Anterior lumbar interbody fusion, Stand-alone cage, Finite element analysis

## Abstract

**Background:**

For anterior lumbar interbody fusion (ALIF), stand-alone cages can be supplemented with vertebral plate, locking screws, or threaded cylinder to avoid the use of posterior fixation. Intuitively, the plate, screw, and cylinder aim to be embedded into the vertebral bodies to effectively immobilize the cage itself. The kinematic and mechanical effects of these integrated components on the lumbar construct have not been extensively studied. A nonlinearly lumbar finite-element model was developed and validated to investigate the biomechanical differences between three stand-alone (Latero, SynFix, and Stabilis) and SynCage-Open plus transpedicular fixation. All four cages were instrumented at the L3-4 level.

**Methods:**

The lumbar models were subjected to the follower load along the lumbar column and the moment at the lumbar top to produce flexion (FL), extension (EX), left/right lateral bending (LLB, RLB), and left/right axial rotation (LAR, RAR). A 10 Nm moment was applied to obtain the six physiological motions in all models. The comparison indices included disc range of motion (ROM), facet contact force, and stresses of the annulus and implants.

**Results:**

At the surgical level, the SynCage-open model supplemented with transpedicular fixation decreased ROM (>76%) greatly; while the SynFix model decreased ROM 56-72%, the Latero model decreased ROM 36-91%, in all motions as compared with the INT model. However, the Stabilis model decreased ROM slightly in extension (11%), lateral bending (21%), and axial rotation (34%). At the adjacent levels, there were no obvious differences in ROM and annulus stress among all instrumented models.

**Conclusions:**

ALIF instrumentation with the Latero or SynFix cage provides an acceptable stability for clinical use without the requirement of additional posterior fixation. However, the Stabilis cage is not favored in extension and lateral bending because of insufficient stabilization.

## Background

**Figure 1:**
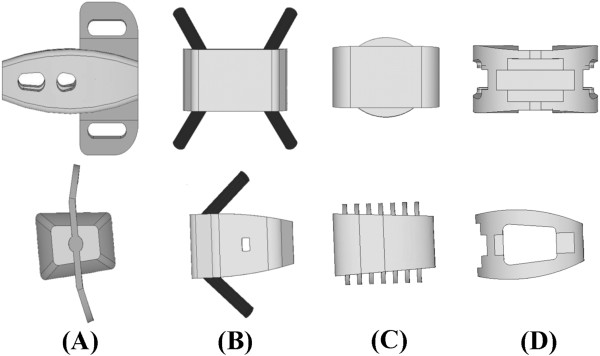
**Front and side views of the four ALIF cages were used in this study. (A)** Latero. **(B)** SynFix. **(C)** Stabilis. **(D)** SynCage-Open.

The lumbar interbody cage is an improvement in spinal fusion which facilitates stabilization of the motion segments and relieves discogenic back pain [1,2]. The common design is either cylindrical or trapezoid in shape and often uses serrated anchorages on the upper and lower surfaces to prevent loosening or subsidence of the cage [3-6]. Stand-alone cages have been used in ALIF treatment and their ability to stabilize the intervertebral motion has been reported to be superior in flexion and bending to extension and rotation [7,8]. In clinical use, the construct stability can be further enhanced by the supplementation of posterior fixation such as pedicle or facet screws [9,10]. However, the significant morbidities of the combined anterior and posterior approaches have been mentioned [11]. These drawbacks may be overcome through the use of newly designed ALIF cages that can be inserted *via* a single anterior or lateral approach with minimal operative morbidity and without causing damage to posterior bony elements and neural, vascular, and muscular tissues [12]. In the past, three stand-alone ALIF cages have been developed, consisting of a trapezoid frame that incorporates the anteriorly stabilizing components (Figure 1). The Latero system (Latero; A-Spine Asia, Taipei, Taiwan) integrates a lateral vertebral plate into the trapezoid frame which is bent to be parallel to the coronal plane. The SynFix system (SynFix; Synthes Spine Inc.,PA, USA) uses four screws to lock the adjacent vertebrae. The Stabilis system (Stabilis; Stryker, Michigan, USA) accommodates a threaded cylinder to anchor the superior and inferior endplates. The stabilizing mechanisms of the Latero plate, SynFix screws, and Stabilis cylinder use the vertebrae as fulcrums to immobilize the bone-cage construct [12,13].

In the literature, comparisons between the different stand-alone ALIF cages have been extensively conducted by the experimental, numerical, and clinical methods [1,2,7]. Using human cadavers as specimens, the three-dimensional stiffness tests in Schleicher’s study [1] demonstrated the effective stabilization ability of the stand-alone SynFix cage in all motion directions. In a previous study by the current authors [14] the numerical results showed no differences of ROM in extension and lateral bending between the Stabilis and SynCage-Open (Synthes Spine, Inc., PA, USA). Except for the differences among cage frames, the stabilizing mechanism might contribute to the postoperative outcome of the stand-alone ALIF [12]. From the biomechanical viewpoint, however, the insertion depth and holding power were quite different between the plate, screw, and cylinder, thus, potentially affecting the stabilizing ability of the stabilizing mechanisms [15].

After surgery, both initial stability and loading transmission of the ALIF level play essential roles in the fusion rate of the instrumented region and the junctional problem of the adjacent region [16]. Especially for osteoporotic bones, bone-cage loosening might result in cage subsidence, interfacial migration, and subsequent nonunion with loss of disc height [9,15]. Jost *et al*. [15] experimentally measured the interfacial strengths of three different bone-cage constructs and declared no significant differences between the threaded Ray cage, the rectangular Brantigan cage, and the porous Contact Fusion cage. Cho *et al.*[2] demonstrated that the stand-alone ALIF cage could assure good clinical results in the surgical treatment of symptomatic lumbar intervertebral foraminal stenosis in a mid-term follow up. To the best of the authors’ knowledge, little study has been dedicated to the detailed investigation of Stabilis performance to date. Moreover, there has been no extensive study devoted to investigate the kinematic and mechanical differences between the plate-, screw-, and thread-type ALIF cages. This constituted the motive of the current study.

Both stress distribution and interfacial micromotion of the bone-cage construct are not easily detectable by experimental methods [17-21]. This study used the finite-element method to evaluate the biomechanical effects of the stand-alone ALIF cages on the kinematic and mechanical behaviors of the adjacent tissues and cages. Three stand-alone ALIF cages (Latero, SynFix, and Stabilis) and one traditional ALIF cage (SynCage-Open) with transpedicular fixation were instrumented into the lumbar models and compared. The detailed investigation focused on the stress distribution and stabilization ability of the Latero plate, SynFix screws, and Stabilis threads. An intact model was used as the comparison baseline and the vertebral strengths were systematically varied. The outcome of this study provides insight into the biomechanical properties of the bone-cage stabilizing mechanism within normal and osteoporotic bones.

## Methods

One intact and four instrumented models of the lumbar spine were constructed in this study. The first was the intact model (INT model) to serve as the comparison baseline. The other four models were instrumented with ALIF cages (Latero, SynFix, Stabilis, and SynCage-Open) and/or transpedicular fixation at the L3-4 level (Figures 1c-d).

### Intact models

**Figure 2:**
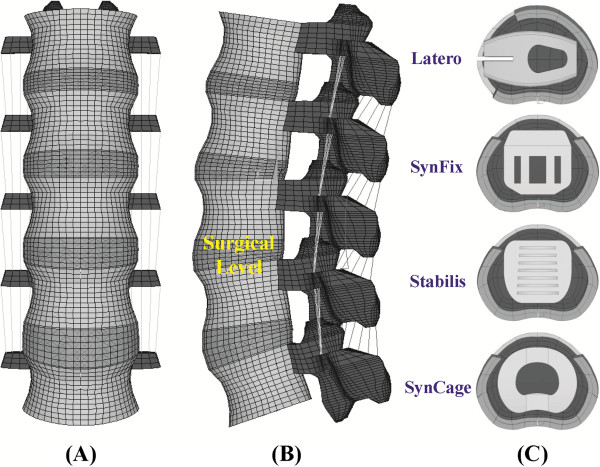
**The lumbar finite-element model used in this study. (A)** Intact model from L1 to L5 levels. **(B)** Instrumented model at the L3-4 level. **(C)** Four ALIF cages instrumented at the L3-4 level.

A three-dimensional nonlinear model of the lumbar spine was constructed from L1 to L5 levels (Figure 2). The lumbar geometry was reconstructed from 1-mm computed tomography scans of a middle-aged male. The CT-scanning images of only one vertebra were used to build the entire lumbar column. The other vertebrae were duplicated and spanned by the intervertebral discs that were manually developed by the CAD software. This makes the vertebral bodies, posterior elements, and associated processes quite similar in shape and size. The pre-procedures of the lumbar FE model were established using ANSYS, Ed.9.0 software (ANSYS Inc., Canonsburg, PA, USA). The INT model was an osseo-ligamentous lumbar spine which includes the vertebrae, intervertebral discs, endplates, posterior elements, and all seven ligaments. The eight-node solid elements were used for modeling the cortical bone, cancellous bone, posterior element, and endplate. The material properties of all tissues were assumed to be homogeneous and transversely isotropic [22]. The intervertebral disc consisted of annulus ground substance, nucleus pulpous, and collagen fibers embedded in the ground substance. The nonlinear annulus ground substance was simulated by using the hyper-elastic Mooney-Rivlin formulation [23]. In the radial direction, twelve double cross-linked fiber layers were defined to decrease elastic strength proportionally from the outermost layer to the innermost. The collagen fibers in different annulus layers were strengthened by the weight factor using the approach from a previous study by the current authors [20]. The weight factors of the elastic modulus were 1.0 at outermost layers, 1–3, 0.9 at layers 4–6, 0.75 at layers 7–9, and 0.65 at the innermost layers, 10–12. The nucleus pulpous was modeled as an incompressible fluid with a bulk modulus of 1666.7 MPa by eight-node fluid elements [22].

No morphological change in vertebrae was simulated in this study. Only decrease in vertebral strength was assumed to model the biomechanical property of the osteoporotic bone. There were two vertebral strengths simulated in this study: normal and osteoporotic models. Compared to the normal bone, “osteoporosis” was defined as a decrease of 66% in the elastic modulus for cancellous bone and a decrease of 33% for the cortical bone [24]. All seven ligaments and collagen fibers were simulated by two-node bilinear link elements with uniaxial tension resistance only, which were arranged in an anatomically correct direction [25]. The cross-sectional areas of each ligament and material properties of the spine were obtained from previous studies (Table 1) [20,22,23,25]. The facet joint was modeled with sliding and non-penetrating behavior using an eight-node surface-to-surface contact element which can slide between three-dimensional target surfaces. The initial gap between a paired facet was kept within 0.5 mm and the coefficient of friction was set at 0.1 [20,22].

**Table 1:** Material properties used in the FE model

**Material**		**Element Type**	**Young’s Modulus (MPa)**		**Area (*mm***^**2**^**)**	**Reference**
			**Poisson’s Ratio**			
Bone			Ex = 11300	*v*xy = 0.484		[22]
Cortical		8-node SOLID185	Ey = 11300	*v*xz = 0.203		
			Ez = 22000	*v*yz = 0.203		
			Gx = 3800			
			Gy = 5400			
			Gz = 5400			
Cancellous		8-node SOLID185	Ex = 140	*v*xy = 0.45		[22]
			Ey = 140	*v*xz = 0.315		
			Ez = 200	*v*yz = 0.315		
			Gx = 48.3			
			Gy = 48.3			
			Gz = 48.3			
Posterior bone		8-node SOLID185	3500	0.25	-	[22]
Disc						[22]
Nucleus pulposus		8-node FLUID80	1666.7	-	-	[22]
Ground Substance		8-node SOLID185	C_10_ = 0.42	-	-	
			C_01_ = 0.105			
Annulus fibers		2-node LINK10				[23]
	Outermost		550	-	0.76	
	Second		495	-	0.5923	
	Third		412.5	-	0.4712	
	Innermost		357.5	-	0.3572	
Cartilaginous Endplates		8-node SOLID185	24	0.4	-	[22]
Ligaments		2-node LINK10				[25]
ALL			7.8	-	24	
PLL			10	-	14.4	
TL			10	-	3.6	
LF			15	-	40	
ISL			10	-	26	
SSL			8	-	23	
CL			7.5	-	30	
Latero (PEEK)		8-node SOLID185	6500	0.2		
Latero-plate (Titanium alloy)		8-node SOLID185	110000	0.3		
SynFix-LR (PEEK)		8-node SOLID185	6500	0.2		
Locking screw (Titanium alloy)		8-node SOLID185	110000	0.3		
Stabilis (Titanium alloy)		8-node SOLID185	110000	0.3		
SynCage-Open (Titanium alloy)		8-node SOLID185	110000	0.3	-	
Pedicle screw (Titanium alloy)		8-node SOLID185	110000	0.3		

*ALL* anterior longitudinal ligament; *PLL* posterior longitudinal ligament; *TL* transverse ligament; *LF* ligamentum flavum; *ISL* interspinous ligament; *SSL* supraspinous ligament; *CL* capsular ligament.

### ALIF models

The Latero cage can be interlocked with a bent lateral plate that is inserted parallel to the coronal plane and into the vertebral bodies, thus preventing cage loosening. The SynFix cage consists of a trapezoid frame incorporated with an anterior plate and screws to stabilize the cage body. The Stabilis cage is a trapezoid frame with a threaded cylinder at the middle region to increase the bone-purchasing ability. Without the serrated anchorages, the final ALIF model of the SynCage-Open cage is further instrumented with bilateral pedicle screws to enhance the construct stability. The models of all cage systems were established by SolidWorks, Ed. 2012 software (SolidWorks Corporation, Concord, MA, USA). The spikes of the Latero cage and the screw threads of the SynFix cage were omitted to simplify numerical calculation (Figure 1).

For ALIF simulation, the L3-4 level of the INT model underwent partial discectomy and total nuclectomy. For the SynFix, Stabilis and SynCage-Open models, the anterior approach was used to remove the anterior longitudinal ligament, anterior and a half inner layer of the annulus, and the entire nucleus pulpous. For the Latero model, the lateral approach was adapted to remove the lateral and a half inner layer of the annulus and the entire nucleus pulpous. All the other ligaments of the three models were preserved.

Accordingly, the Latero, SynFix, Stabilis, and SynCage-Open peek cages were modeled and inserted into the L3-4 level. The friction coefficient of the bone-cage interfaces was 0.8 to mimic a serrated surface for the initial stability of the trapezoid frame [14]. The SynCage-Open model was supplemented with bilateral transpedicular fixation. In the SynCage-Open and SynFix models, the pedicle screw (6-mm diameter) and locking screw (4-mm diameter) were respectively modeled with three-dimensional beam elements. The bone-screw interfaces were assumed to be fully bonded to simulate intimate bone-screw purchase. The numbers of elements and nodes were 98,988/135,786, 112,087/253,492, 114,849/162,169, and 106,436/180,875 for the Latero, SynFix, Stabilis, and SynCage-Open models, respectively. The material properties of the ALIF cages, supplementary components, and transpedicular fixator were listed in Table 1.

### Finite-element analyses

Only vertical compression onto the lumbar top can potentially lead to excessive movement of the lumbar column [16]. In this study, the follower load was used to constrain each motion segment of the lumbar model with the two-node truss elements that induce contractions for a given temperature change [26]. In principle, the ideal follower load remains tangent to the spine curve, and each spinal segment is loaded in nearly pure compression without artifact motions. Using the trial-and-error method, the attached points of truss elements were modified to optimize the follower load path approximated through the instantaneous center of rotation at each motion segment [27]. This study cautiously decreased the temperature of the truss elements to produce a 400-N follower load for minimizing the ROM of each motion segment (<0.2°). Subsequently, a 10-Nm moment was applied to the lumbar top to simulate flexion-extension, left/right lateral bending, and left/right axial rotation, respectively. During simulation, the bottom of the L5 vertebral body was fixed completely. The comparison indices were intersegmental ROM, facet force, and stresses of annulus, implant, and endplate. The annulus stress is the stress of the ground substance. From the biomechanical viewpoint, the annulus fibers are mainly responsible for the tension. However, the ground substance can bear the various loads that always exist within the intervertebral discs. Previous *in vitro* studies demonstrated that a disc may prolapse under certain load combinations of flexion, lateral bending, axial rotation, and axial compression [28-30]. Only the stress of the annulus fibers cannot provide the sufficient information about the disc loads. Consequently, this study uses the stress of the ground substance as the index of the disc prolapse and herniation.

## Results

**Figure 3:**
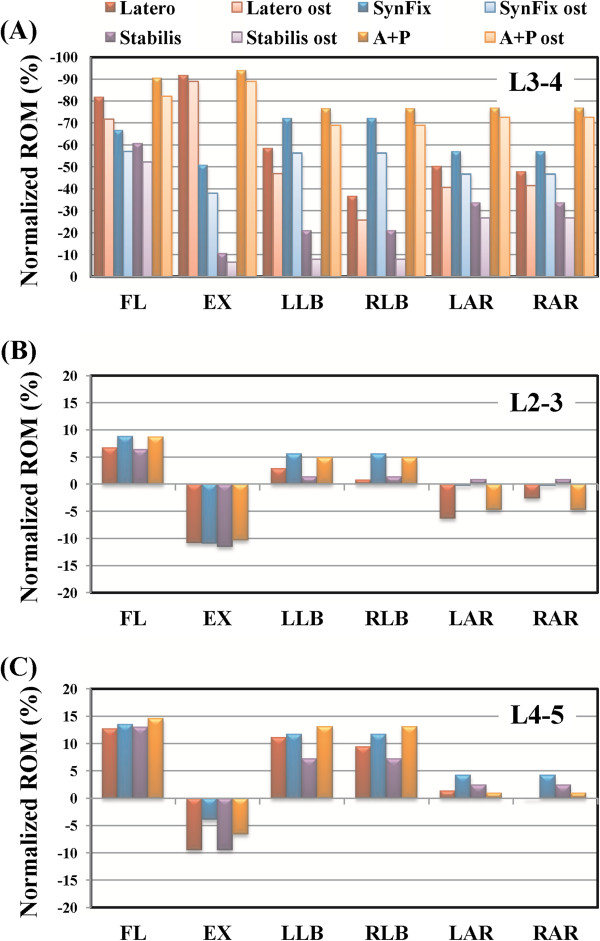
**Comparison of the normalized intersegmental ROM among all models under six motions. (A)** Surgical level.**(B-C)** Adjacent levels.

**Figure 4:**
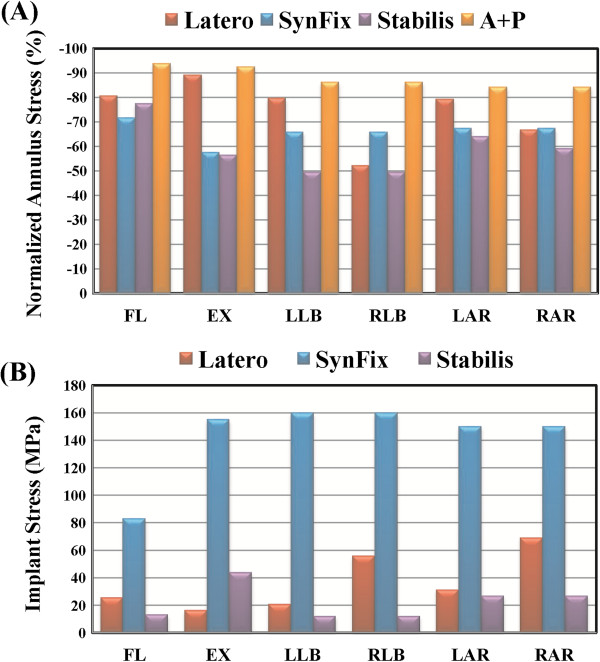
**Stress comparison of the normalized stress among all models under six motions. (A)** Annulus stress. **(B)** Implant stress.

**Figure 5:**
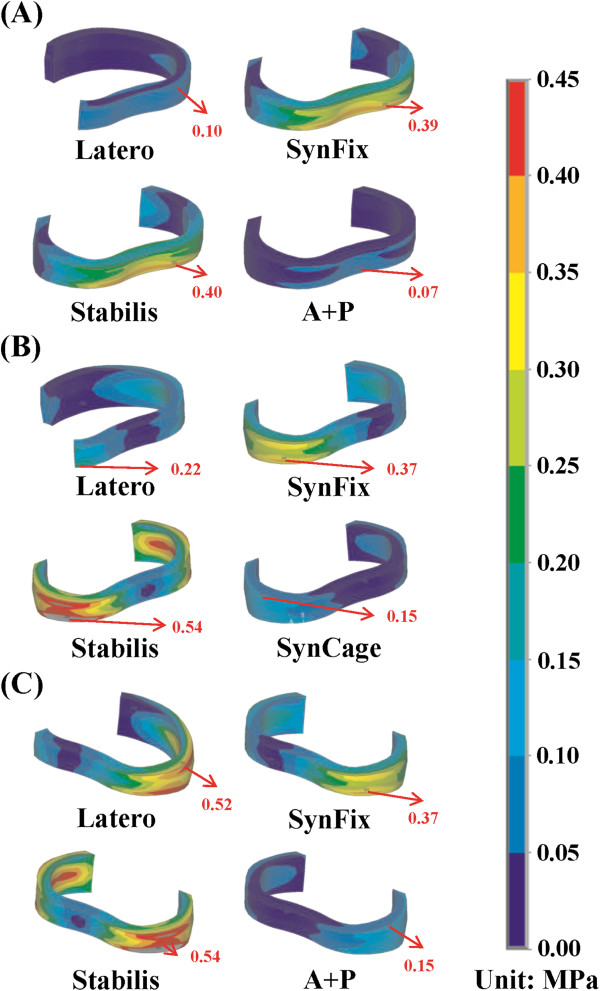
**Stress distribution of the ground substance at the surgical level for all models. (A)** Extension **(B)** Left lateral bending. **(C)** Right lateral bending.

**Figure 6:**
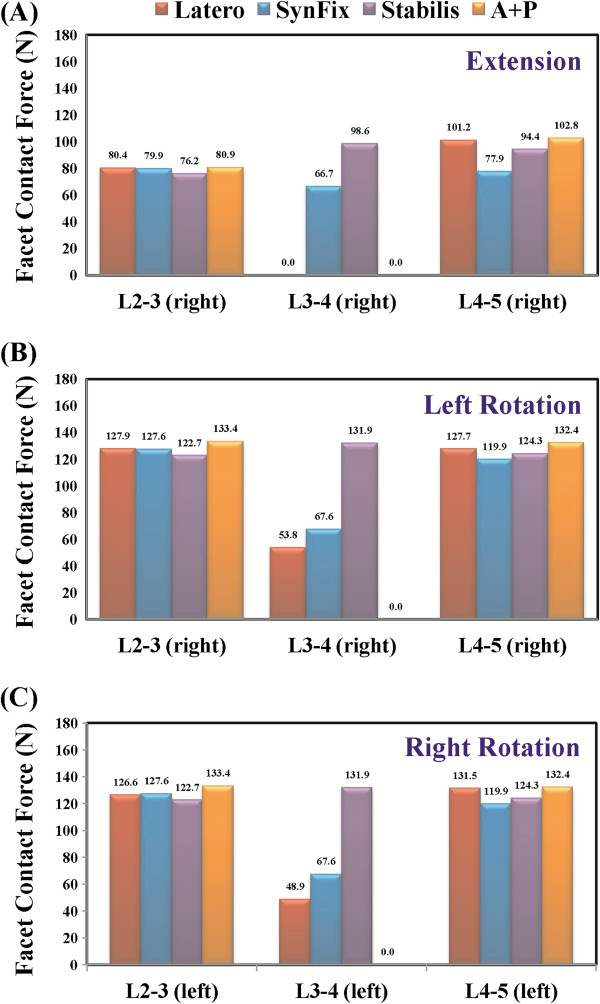
**Comparison of facet contact force among all models. (A)** Extension. **(B)** Left axial rotation. **(C)** Right axial rotation. Middle bars are the surgical level (L3-4); left and right bars are the adjacent levels (L2-3 and L4-5).

**Figure 7:**
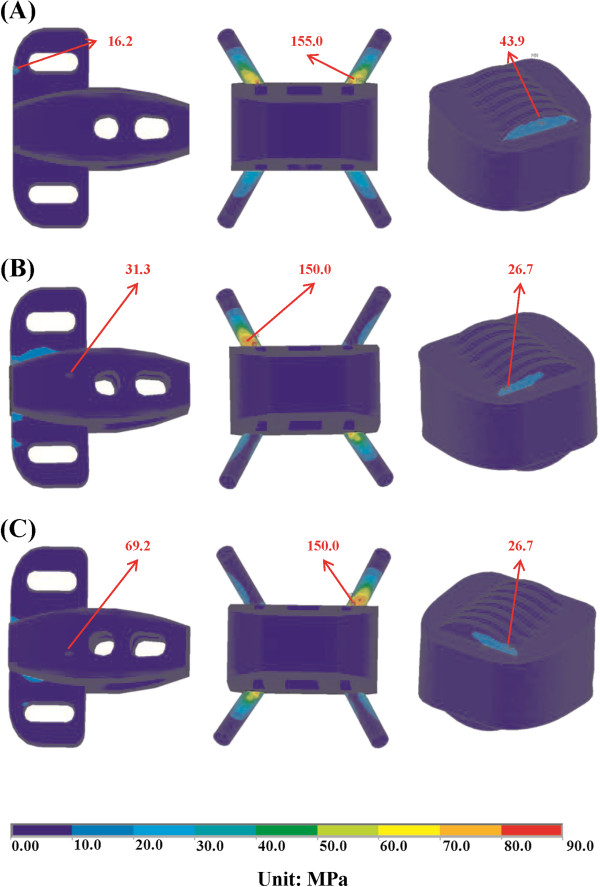
**Distribution of implant stress for the Latero, SynFix, and Stabilis models. (A)** Extension. **(B)** Left lateral bending.**(C)** Right lateral bending.

**Figure 8:**
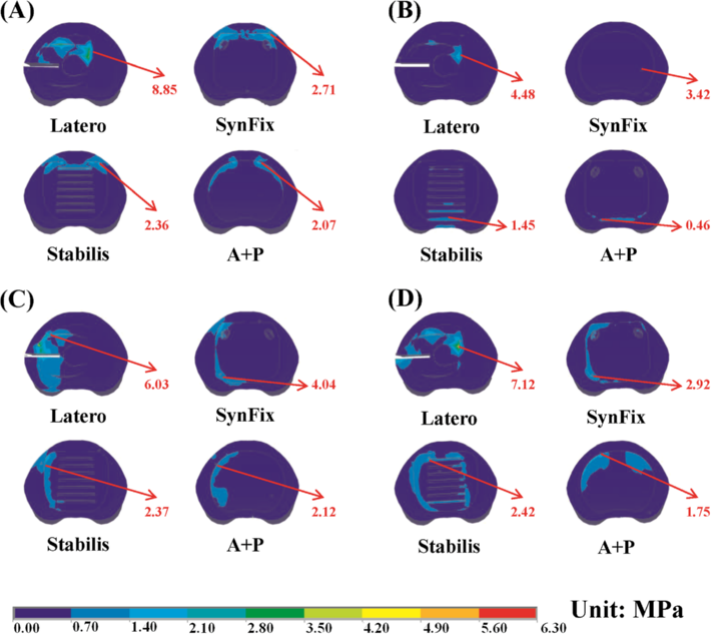
**Distribution of endplate stress on the upper surface of the L4 vertebra for all models. (A)** Flexion. **(B)** Extension. **(C)** Left lateral bending. **(D)** Left axial rotation.

This study used four parameters as the comparison indices, including intersegmental ROMs, annulus stress, endplate stress, and facet contact forces (Figures 3, 4,5, 6, 7 and 8). There were five models: one intact (INT), 3 stand-alone ALIF cages (Latero, SynFix, and Stabilis), and one established fixation (A + P: SynCage with transpedicular fixation). The ROM comparison of the instrumented models with normal and osteoporotic bone was normalized by the corresponding value of the intact model.

### Convergence and validation of the intact model

The convergence test consisted of three mesh qualities: 4,750 elements / 4,960 nodes for the coarse model, 27,244 elements / 30,630 nodes for normal model, and 84,594 elements / 94,162 nodes for the finest model. For the finest mesh quality, the changes in total ROM were respectively within 1.03% in flexion (< 0.2°), 4.39% in extension (< 0.5°), 0.01% in torsion (<0.2°), and 0.001% in lateral bending (< 0.1°). Consequently, this study used the finest model to evaluate the biomechanical behaviors of the INT and ALIF models under four physiological motions.

For model validation, the ROM changes in five levels of the INT model were compared with the experimental results of Rohlmann’s study [27]. Under 3.75- and 7.5-Nm moments with 150-N preload, a previous study [31] by the current authors showed data of the current INT model within the extreme values of Rohlmann’s results. Under a 10-Nm moment with 150 N preload, however, the predicted ROMs of this study were 6° to 11° less than those of the *in vitro* tests under flexion. This might be explained by the differing preload applications of the current (pressure preload) and earlier *in vitro* tests (vertical preload) [12,14]. The pressure preload applied a compressive force of 150 N that was always perpendicular to the lumbar top. However, the compression of the vertical preload was consistently orthogonal to the horizontal plane during lumbar motion. Consequently, the pressure preload resulted in a much lower bending moment compared with the vertical preload. In torsion, the facet contact force of the INT model ranged between 121 to 130 N and the values were within the ranges of earlier studies [25,32]. This indicated that the INT model was well verified for further simulation of the four ALIF models. In total, there were nine models (one intact, four instrumented × two bones) and six motions simulated in this study.

### Intersegmental ROM at the surgical and adjacent levels

Referring to Panjabi’s ALE formula [33], the restricted ROM of the instrumented model was compared with the corresponding ROM value of the INT model (Figure 3).

The A + P model had the maximal capability in restricting ROM from −76.5% to −93.8% in all motions. The Latero model performed ROM control similar to that of A + P, and superior to that of the SynFix model in flexion and extension. Moreover, the Latero model performance was similar to that of the SynFix model in bilateral axial rotation and left lateral bending, but inferior in right lateral bending. The asymmetrical design of the Latero model with the vertebral plate placed on the left side explained the different behaviors in left *versus* right lateral bending. The Stabilis model had the lowest values in all motions, especially in controlling lateral bending (−21.0%) and extension (−10.6%). This finding is consistent with the result of the previous study by the current authors [14].

For the osteoporotic lumbar, the percentages of restricted ROM at the surgical level for all models were also shown in Figure 3. Under the osteoporotic condition, the percentages of restricted ROM consistently decreased for all models. The percentages of change rate were 2.7% ~ 10.8% in the Latero, 9.7% ~ 15.9% in the SynFix, 4.4% ~ 13.0% in the Stabilis, and 4.8% ~ 8.2% in the A + P models. The maximal change rate was around 10% in the Latero (10.8%) and A + P (8.2%) models, and slightly higher in the SynFix (15.9%) and Stabilis (13.0%) models. Under the osteoporotic condition, the A + P model still had 70% of ROM control; while the Latero model had weaker control in right lateral bending (−25.9%, *versus −*36.7% in normal bone), the SynFix model had weaker control in extension (−38.0%, *versus −*50.7% in normal bone), and the Stabilis model had even weaker control in extension (−6.2%) and lateral bending (−8.0%).

ROM control of the adjacent levels under normal and osteoporosis conditions for all models were shown in Figures 3a-c. Under both conditions, the percentages of ROM change rate were less than 12.3% at L2-3, and less than 15.8% at the L4-5 level as compared to the INT model. The difference of ROM change rates was small at the adjacent levels of each model under normal and osteoporotic conditions.

### Annulus stress

Referring to Panjabi’s formula [33], the normalized percentages of the maximum annulus stress at the surgical level of all models were shown in Figure 4a. At the surgical level, the normalized annulus stress and intersegmental ROM can be well correlated for each model. The Latero model had maximum annulus stress similar to the A + P model, and was superior to the SynFix model in flexion and extension. It was also similar to the SynFix model in bilateral axial rotation and left lateral bending, but inferior in right lateral bending.

Under the normal condition, the annulus stress distribution of the four instrumented models was shown in extension, right and left lateral bending (Figures 5a-c). The Latero had annulus stress distribution similar to the A + P model in extension (Figure 5a), similar to the Stabilis model in right lateral bending (Figure 5b), and similar to the SynFix model in left lateral bending (Figure 5c). The Stabilis model showed the highest annulus stress in all motions, with the stress being concentrated at the posterior annulus in extension (Figure 5a) and at the lateral annulus in bilateral lateral bending (Figures 5b, c). In contrast, the annulus stress was more evenly distributed in the other three models. However, at the adjacent L2-3 and L4-5 levels, there was no obvious difference in annulus stress distribution among the four instrumented models.

### Facet contact force

The facet contact forces at the surgical and adjacent levels of all models are shown in extension and bilateral rotation (Figures 6a-c). The A + P model had nearly zero facet contact force at the surgical level in all motions, because the relative motions of the facets joint were restricted by the pedicle screw. In extension, the Latero model could control most of extension (−91.6%) and there was little force shifted to the facet joints (Figure 6a). In bilateral axial rotation, the facet contact force of the Latero and SynFix models were similar, because both devices had similar capability in controlling axial rotation (Figures 6b-c). The highest values of facet contact force at the surgical level of the Stabilis model could be explained by the poorer control of extension and axial rotation. At the adjacent levels, there were small differences (<15.0 N) of facet contact force among the four models in extension and axial rotation (Figures 6a-c; left: left facet and right: right facet).

### Implant stress

The maximum stresses sustained by the integrated parts of three stand-alone cages were shown in Figure 4b. The locking screws of the SynFix model had higher stress than the vertebral plate of Latero and the threaded cylinder of Stabilis models in all motions. The ratios of maximum stresses at the integrated parts among Latero, SynFix and Stabilis models were 1: 9.6: 2.7 in extension, 1: 2.9: 0.2 in right lateral bending, and 1: 2.2: 0.4 in right axial rotation, respectively. The maximum stress at the integrated parts of the Latero model was similar to that of the Stabilis model in left lateral bending and left axial rotation.

The stress distribution at the integrated part of the three stand-alone models was shown in extension, right lateral bending, and right axial rotation (Figures 7a-c). In these three motions, the stress was concentrated at the base of the locking screws in the SynFix model; while the stress was evenly distributed over the vertebral plate in the Latero model and the threaded cylinder in the Stabilis model.

### Endplate stress

Since cage subsidence most commonly occurs at the lower endplate of the upper vertebra [2], only the maximum stress on the L3 endplate was calculated. All models consistently showed that the maximum stress occurs at the top surface of the L3 vertebra (Figure 8). The adjacent endplate of the Latero model was more stressed than the other models in axial rotation. Among all motions, the L3 endplate was most stressed in flexion at the SynFix model. In extension, the stress ratios of the contact surface on L3 between the three stand-alone cage models in extension equal to 1:1.07:1.15 in Latero, SynFix, and Stabilis, respectively. The aforementioned stress ratios were 1:1.47:0.53, 1:0.48:0.53 and 1:0.31:0.34 in flexion, left axial rotation and right axial rotation, respectively. The highly stressed endplate of the Latero model in axial rotation might be attributed to the smaller contact surface of the Latero cage.

## Discussion

Nowadays, the interbody fusion cage with transpedicular fixation has been established as the standard for lumbar degenerative disorders due to its high fusion rate. However, there have been concerns about approach- and device-related issues as well as adjacent segment disease (ASD). ASD is an important long-term issue in lumbar fusion. The literature has shown the incidence of ASD when using pedicle screws is much higher than that for ALIF, where the incidence of ASD after anterior fusion is similar to that under natural conditions [34]. Recently, surgical trends have shifted using minimally invasive techniques through alternative approaches and reduced loads of fixation devices to mitigate the issues of concern. Examples of such techniques are anterior stand-alone cages coupled with self-stabilizing mechanisms, such as the Latero plate, SynFix screw, and Stabilis cylinder, which eliminate the need of posterior fixation (Figure 1). Despite the claimed improvements in construct stability, anterior access has disadvantages, including risk of vascular injury and sacrificing of the anterior longitudinal ligament [35].

In this study, the results of ROM control showed that the A + P model had the best overall performance and the Stabilis model had the weakest ROM control. Both Latero and SynFix models were shown to have >50% of ROM control in all motions except for that of the Latero model, which demonstrated a discrepant control of left (−59%) and right lateral bending (−37%). The asymmetrical design of the Latero cage and its instrumentation in the left side of interspace may explain the different left and right bending behaviors. In practice, this discrepancy might be compensated for by adding bone grafts or elongating the cage to cover the right side of the interspace. Various biomechanical studies have shown that stand-alone cages lack control of extension and axial rotation but not of flexion and lateral bending [7]. The Latero model (−92%) had comparable control in extension as the A + P model (−94%) and was significantly superior to the SynFix model (−51%). This could be explained by the force vector of extension which was directly perpendicular to the Latero plate. This also accounts for the excellent control of extension and axial rotation by the Latero plate in comparison to the other counterparts (Figure 3).

Under osteoporotic conditions, all models lost a little ROM control at the surgical level, ranging from 3% to 16% (Figure 3). In real life with osteoporosis, the screw-based devices (SynFix and A + P) potentially exhibit screw loosening after cyclic loading. Although the rigidity of the screw-bone interface may be enhanced by injecting cement, there is the possibility of cement-related hazards. In Stabilis, the bony endplates might be compromised by threading in the cylindrical cage, and a higher incidence of cage subsidence than in other cages has been reported [2]. The Latero plate utilizes a stabilizing mechanism which is dissimilar from those of other devices; it remains to be proved clinically whether the Latero model behaves differently in an osteoporotic lumbar. At the adjacent levels, their segmental ROMs were indeed changed when compared with the intact state. However, the changes were small among these four models, even in the stiffest A + P model which is known to have a higher incidence of ASD at long-term follow up. This finite-element study cannot reflect the consequences of clinical cyclic loading, it can only reveal the ROM changes of adjacent levels.

The varying design concepts of the integrated parts in the self-stabilizing cages not only affect their ROM control, but also influence the stress distribution on the device and the adjacent tissues. In this study, the stress distribution on the Latero plate was much lower and more evenly distributed than that of the SynFix screws (Figure 7). The SynFix screws sustained 3.75 to 9.57 times greater stresses than the Latero plate, with the stress concentrated at the cage-screw junctions. For the Latero and SynFix models, the difference in modes and amounts of distributed stress might be explained by the different configurations of the stabilizing components: sizes, contact areas, and the distance to the center of rotation. In cyclic loading, the Latero plate might be less likely to have fatigue failure than the SynFix screws. The cylindrical cage of the Stabilis model sustained the lowest stress as a result of its relatively suboptimal ROM control.

Stress distribution on the vertebra-cage interface represents vertical compression force exerted on the bony endplate. The Latero model exerted the highest stress on the L4 endplate compared to the other models (Figure 8). This might be due to the stress not being fully shielded by the intervertebral plate and being redistributed by the plate and transmitted onto the L4 endplate. Comparatively, the stress was shielded by the screws of the SynFix and A + P models and thus less stress was distributed on the endplate. In the Stabilis model, because of its relatively poor ROM control, the distributed stress on the endplate was less than in the other three models. There were two biomechanical implications in the Latero model, which had higher vertical load on the L4 endplate than the other counterparts. The first implication indicates that higher vertebral stress may increase the incidence of cage subsidence particularly in suboptimal bone density. On the other hand, unshielded vertical load may be beneficial for graft consolidation according to Wolff's law.

After interbody fusion, abnormally high transmission of loads to the facet joints may ultimately result in arthritic changes. At the surgical level, when comparing the three stand-alone cage models, the Latero model was shown to have the lowest values of facet contact force in bilateral rotation and was near absent in extension (Figure 6). The facet joints at the surgical level seemed to be relatively well protected in the Latero model in comparison to the SynFix and Stabilis models. At the adjacent levels, there were few differences of facet contact force among all four instrumented models. This indicates that the reasons for the degenerative facet joints at the adjacent levels might be attributed to the other clinical factors.

Distribution of annulus stress can provide the clinical implication that higher stress may result in annulus disruption and disc herniation [28-30]. At the surgical level for the four instrumented models, the distribution of annulus stress was well correlated inversely to their ROM control (Figures 3, 4a). At right lateral bending, the highest annulus stress was found in the Latero model, where it was concentrated at the right annulus. This corresponded to the relatively inferior control of the Latero due to its asymmetrical design. The posterior and left sides of annulus in the Stabilis model sustained the highest stress at extension and lateral bending, which manifested in its inferior control of those moments.

## Conclusions

There were several limitations inherent in this study. Bone fusion was not included and only the effect of the initial stability was considered for the stand-alone cages. Degenerative discs are common in most patients with ALIF surgery; however, it is challenging in modeling to assign material properties to various grades of degenerative discs, such as delamination, dehydration, or reduced disc height. These variations of the lumbar tissues were not included in this study. In addition, the geometry of implants was somewhat simplified for mesh modeling. This study was not concerned with the effect of bone ingrowth into the cage and ligament pretension after inserting the implants. The stabilizing behaviors of trunk muscles were mimicked by the follower load that has been extensively used in related *in vitro* tests [16,27]. However, the real situations of muscle contraction and complicated external load conditions *in vivo* were not investigated. The fatigue failure of the cages’ components was not studied in the static simulation and the current authors we recommend that they should be evaluated by experimental or clinical observation.

In conclusion, this study extensively compared the stabilizing mechanisms of three stand-alone ALIF cages. The vertebral plate of the Latero model provided sufficient ability in stabilizing intersegmental motions: it was comparable to the A + P model in flexion and extension, comparable to the SynFix in bilateral axial rotation and left lateral bending, and inferior but compensable in right lateral bending. In contrast, the Stabilis model was less favorable due to its poorer control in extension and lateral bending. Further experimental and clinical studies should be conducted to validate the numerical observations.

## Abbreviations

ALIF: Anterior lumbar interbody fusion; INT: Intact model; A + P: Model, SynCage with transpedicular fixation; ROM: Range of motion; FL: Flexion; EX: Extension; LLB: Left lateral bending; RLB: Right lateral bending; LAR: Left axial rotation; RAR: Right lateral bending.

## Competing interests

The authors declare that they have no competing interests.

## Authors’ contributions

MCC participated in the study design, in collecting the data, the statistically analyses and drafting of the manuscript. SHC, JFL and CHH participated in the study design. SCL advised and assisted drafting of the manuscript. All authors read and approved the final manuscript.

## Pre-publication history

The pre-publication history for this paper can be accessed here:

http://www.biomedcentral.com/1471-2474/14/281/prepub
